# Crystal structures of *trans*-di­aqua­(3-*R*-1,3,5,8,12-penta­aza­cyclo­tetra­deca­ne)copper(II) isophthalate hydrates (*R* = benzyl or pyridin-3-ylmethyl)

**DOI:** 10.1107/S2056989019008387

**Published:** 2019-06-21

**Authors:** Irina L. Andriichuk, Liudmyla V. Tsymbal, Vladimir B. Arion, Yaroslaw D. Lampeka

**Affiliations:** a L. V. Pisarzhevskii Institute of Physical Chemistry of the National Academy of Sciences of Ukraine, Prospekt Nauki 31, 03028 Kiev, Ukraine; b Institute of Inorganic Chemistry of the University of Vienna, Wahringer Str., 42, 1090 Vienna, Austria

**Keywords:** crystal structure, aza­macrocyclic ligand, aza­cyclam, copper, isophthalic acid, hydrogen bonds

## Abstract

The asymmetric units of the title compounds, *trans*-di­aqua­(3-benzyl-1,3,5,8,12-penta­aza­cyclo­tetra­decane-κ^4^ *N* ^1^,*N* ^5^,*N* ^8^,*N* ^12^)copper(II) isophthalate monohydrate, [Cu(C_16_H_29_N_5_)(H_2_O)_2_](C_8_H_4_O_4_)·H_2_O, (I), and *trans*-di­aqua­[3-(pyridin-3-ylmeth­yl)-1,3,5,8,12-penta­aza­cyclo­tetra­decane-κ^4^ *N* ^1^,*N* ^5^,*N* ^8^,*N* ^12^]copper(II) iso­phthalate 0.9-hydrate, [Cu(C_15_H_28_N_6_)(H_2_O)_2_](C_8_H_4_O_4_)·0.9H_2_O, (II) consist of one di­aqua macrocyclic cation, one di­carboxyl­ate anion and uncoordinated water mol­ecule(s). In each compound, the metal ion is coordinated by the four secondary N atoms of the macrocyclic ligand and the mutually *trans* O atoms of the water mol­ecules in a tetra­gonally distorted octa­hedral geometry. The average equatorial Cu—N bond lengths are significantly shorter than the average axial Cu—O bond lengths [2.020 (9) *versus* 2.495 (12) Å and 2.015 (4) *versus* 2.507 (7) Å for (I) and (II), respectively]. The coordinated macrocyclic ligand in the cations of both compounds adopts the most energetically favorable *trans*-III conformation. In the crystals, the complex cations and counter-anions are connected *via* hydrogen-bonding inter­actions between the N—H groups of the macrocycles and the O—H groups of coordinated water mol­ecules as the proton donors and the O atoms of the carboxyl­ate as the proton acceptors. Additionally, as a result of O—H⋯O hydrogen bonding with the coordinated and water mol­ecules of crystallization, the isophthalate dianions form layers lying parallel to the (01) and (100) planes in (I) and (II), respectively.

## Abstract 2

The complex cations of the title compounds, (I) and (II), contain tetra­gonally distorted CuN_4_O_2_ octa­hedra with four N atoms of the aza­macrocyclic ligand in the equatorial planes and two O atoms of the water mol­ecules in the axial positions. In the crystals, the isophthalate counter-ions form layers as a result of O—H⋯O hydrogen bonds with the water mol­ecules, which are pillared with the macrocyclic cations and lie parallel to the (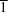
01) and (100) planes in (I) and (II), respectively.

## Chemical context   

Transition-metal complexes of the versatile macrocyclic 14-membered tetra­amine ligand cyclam (cyclam = 1,4,8,11-tetra­aza­cyclo­tetra­deca­ne) are popular metal-containing building units for the construction of metal–organic frameworks (MOFs) possessing many promising applications (Lampeka & Tsymbal, 2004 ▸; Suh & Moon, 2007 ▸; Suh *et al.*, 2012 ▸; Stackhouse & Ma, 2018 ▸; Lee & Moon, 2018 ▸). Such an inter­est is explained by the exceptionally high thermodynamic stability and kinetic inertness of these species (Melson, 1979 ▸; Yatsimirskii & Lampeka, 1985 ▸), implying a preservation of their structural features (equatorial arrangement of the macrocycle in the coordination sphere of the metal ion, availability of two *trans* vacant sites in the axial positions suitable for coordination of bridging ligands), thus making the architecture of MOFs more predictable. The complexes of *N* ^3^,*N* ^10^-disubstituted di­aza­cyclam (di­aza­cyclam = 1,3,5,8,10,12-hexa­aza­cyclo­tetra­deca­ne), readily obtainable *via* template-directed Mannich condensation of bis­(ethyl­enedi­amine) complexes with formaldehyde and primary amines (Costisor & Linert, 2000 ▸), also represent widespread systems in this kind of investigations. At the same time, the complexes of *N* ^3^-substituted aza­cyclam (aza­cyclam = 1,3,5,8,12-penta­aza­cyclo­tetra­deca­ne) – a middle member of this series of ligands – have attracted considerably less attention, presumably because of the necessity of using a more sophisticated non-cyclic precursor, *i.e*. 3,7-di­aza­nonane-1,9-di­amine, in the Mannich condensation (Rosokha *et al.*, 1993 ▸).
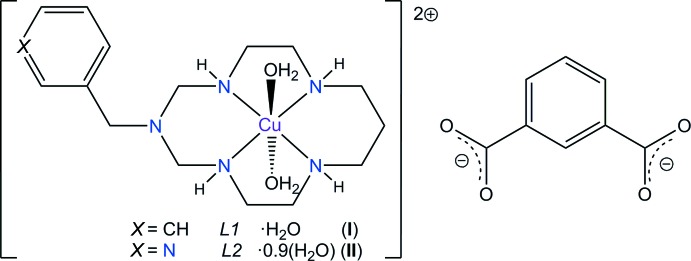


Though the isophthalate (1,3-benzene­dicarboxyl­ate) dianion is often used as bridging ligand in the construction of MOFs, a very limited number of its compounds with aza­macrocyclic cations have been described to date and all they are complexes of the Ni^II^ ion.

Herein, we describe the syntheses and crystal structures of the title Cu^II^ complexes with aza­cyclam ligands and an iso­ph­thalate dianion, namely, *trans*-di­aqua­(3-benzyl-1,3,5,8,12-penta­aza­cyclo­tetra­decane-κ^4^ *N^1^,N^5^,N^8^,N^12^*)copper(II) iso­ph­thal­ate hydrate, [Cu(*L1*)(H_2_O)_2_](ip)·H_2_O, (I), and *trans*-di­aqua­[3-(pyridin-3-ylmeth­yl)-1,3,5,8,12-penta­aza­cyclo­tetra­decane-κ^4^ *N* ^1^,*N* ^5^,*N* ^8^,*N* ^12^]copper(II) isophthalate 0.9-hydrate, [Cu(*L2*)(H_2_O)_2_](ip)·0.9(H_2_O), (II).

## Structural commentary   

**Figure 1:**
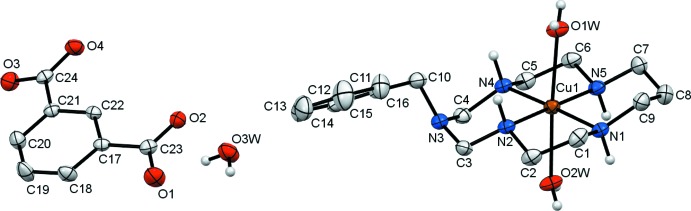
View of the asymmetric unit of (I), showing the atom-labelling scheme, with displacement ellipsoids drawn at the 30% probability level. H atoms attached to carbon atoms have been omitted for clarity.

**Figure 2:**
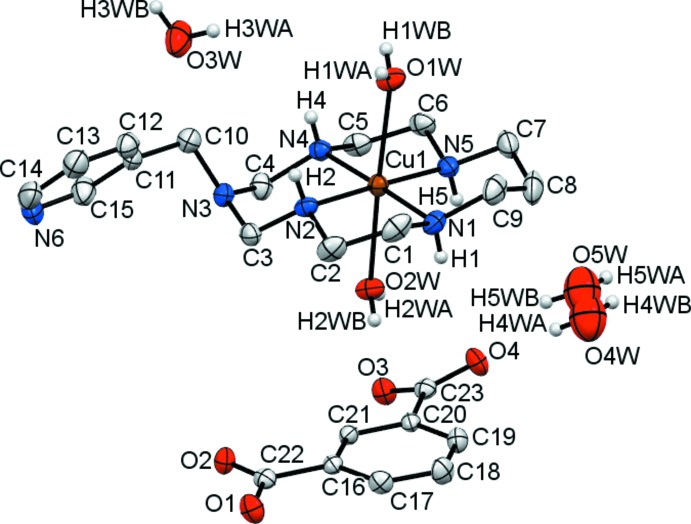
View of the asymmetric unit of (II), showing the atom-labelling scheme, with displacement ellipsoids drawn at the 30% probability level. H atoms attached to carbon atoms have been omitted for clarity.

Each Cu^II^ ion in the complex cations in the title compounds (I) and (II) is coordinated in the equatorial plane by four secondary amine N atoms of the aza­macrocyclic ligand in a square-planar fashion, and by two O atoms from the water mol­ecules in the axial positions, resulting in a tetra­gonally distorted octa­hedral geometry (Table 1 ▸, Fig. 1 ▸ and Fig. 2 ▸).

The average equatorial Cu—N bond lengths are significantly shorter than the average axial Cu—O bond lengths [2.020 (9) *versus* 2.495 (12) Å for (I) and 2.015 (4) *versus* 2.507 (7) Å for (II)], which can be attributed to a large Jahn–Teller distortion. The Cu^II^ ions are displaced from the nearly planar (r.m.s. deviations less than 0.01 Å) mean planes of the N_4_ donor atoms towards the O1*W* water mol­ecule by 0.024 and 0.033 Å in (I) and (II), respectively. Both coordinated macrocyclic ligands adopt the most energetically favourable *trans*-III (*R,R,S,S*) conformation (Bosnich *et al.*, 1965 ▸) with the five-membered chelate rings in *gauche* [bite angles 86.28 (1) for (I) and 86.30 (7)° for (II)] and six-membered chelate rings in *chair* [bite angles 93.7 (2) for (I) and 93.7 (9)° for (II)] conformations. The methyl­ene group of the substituent at the non-coordinated nitro­gen atoms N3 in the six-membered chelate rings is axially oriented and the sum of the C—N—C angles around these atoms [345.6 and 348.1° for (I) and (II), respectively] indicates their partial *sp^2^* character (Tsymbal *et al.*, 2019 ▸).

The isophthalate dianions in the title compounds counterbalance the charge of the complex cations. One carb­oxy­lic group of the isophthalate (O1/O2/C) is nearly coplanar with the mean plane of the aromatic fragment [dihedral angles being 2.4 (3) and 3.6 (4)° in (I) and (II), respectively], while the second (O3/O4/C) is tilted by 11.6 (3) and 21.1 (4)° in (I) and (II), respectively. The C—O bond lengths in the carb­oxy­lic groups are nearly equal, thus indicating essential electron delocalization.

Among the water mol­ecules of crystallization, O3*W* in (I) is fully occupied, while that in (II) has a site occupancy of 50%. Additionally, two positions for disordered water mol­ecules (O4*W* and O5*W*), each with 20% population, were found in (II). Because of their low partial population, these were not considered further in the analysis of the hydrogen-bonding network.

## Supra­molecular features   

**Figure 3:**
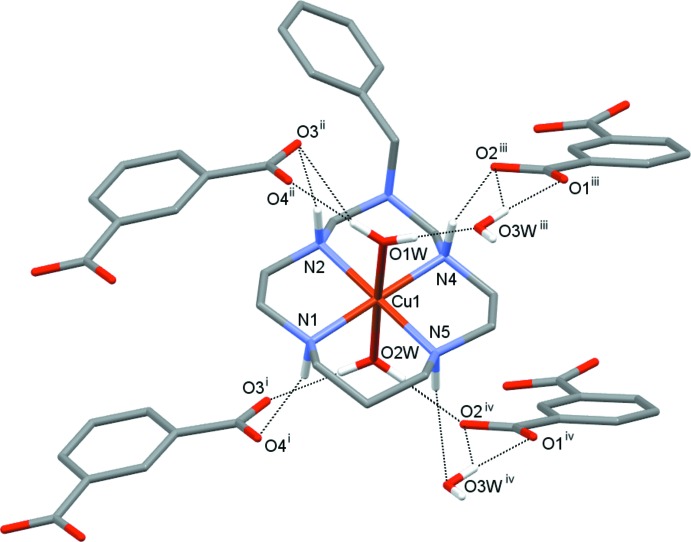
Nearest surrounding of the macrocyclic cation in (I) formed by hydrogen bonding (dashed lines). [Symmetry codes: (i) *x* – 1, *y* + 1, *z*; (ii) *x*, *y* + 1, *z*; (iii) –*x* + 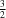, *y* + 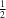, –*z* + 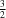; (iv) –*x* + 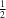, *y* + 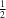, –*z* + 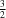.]

Three secondary amino groups of the coordinated macrocycle in (I) act as proton donors by the formation of N—H⋯O hydrogen bonds with the carb­oxy­lic groups of three different adjacent anions, while the fourth group forms hydrogen bond with the water mol­ecule of crystallization O3*W* (Fig. 3 ▸, Table 2 ▸). In turn, the coordinated water mol­ecules donate protons to the carb­oxy­lic group of the anion {bifurcated hydrogen bonding O1*W*—H1*WB*⋯[O3,O4(*x*, *y* + 1, *z*)] and O2*W*—H2*WA*⋯O2(−*x* + 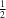, *y* + 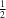, −*z* + 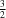) and O2*W*—H2*WB*⋯O3(*x* − 1, *y* + 1, *z*)}, as well as to the O3*W* mol­ecule [O1*W*—H1*WA*⋯O3*W*(−*x* + 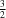, *y* + 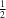, −*z* + 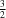)]. Additionally, the uncoordinated water mol­ecule O3*W* acts as a proton donor by the formation of bifurcated O3*W*—H3*WB*⋯(O1,O2) and O3*W*—H3*WA*⋯O1(−*x* + 1, −*y* + 1, −*z* + 1) hydrogen bonds.

**Figure 4:**
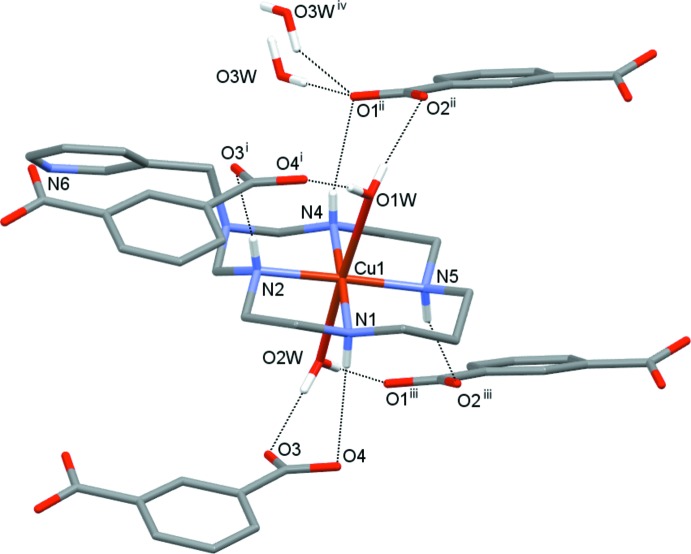
Nearest surrounding of the macrocyclic cation in (II) formed by hydrogen bonding (dashed lines). [Symmetry codes: (i) *x* + 1, *y*, *z*; (ii) *x* + 1, −*y* + 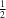, *z* + 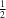; (iii) *x*, −*y* + 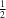, *z* + 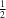; (iv) –*x* + 2, –*y*, –*z* + 1.] The contact C1—H1*B*⋯N6 (−*x* + 1, *y* + 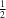, −*z* + 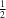) is not shown.

The hydrogen-bonded network in (II), though slightly different, has much in common with that in (I). In particular, all secondary amino groups of the macrocycle form N—H⋯O hydrogen bonds acting as proton donors with the carb­oxy­lic groups of four different adjacent anions (Fig. 4 ▸, Table 3 ▸). Each coordinated water mol­ecule, as well as the water mol­ecule of crystallization O3*W*, donates protons to two carb­oxy­lic groups of different isophthalate anions. Additionally, in the crystal of (II) there are a number of C—H⋯O and C—H⋯N contacts between the methyl­ene and methine groups of the macrocyclic ligand and oxygen atoms of carb­oxy­lic groups, the water mol­ecule O3*W* and atom N6 of the substituent in the neighbouring macrocycle (Table 3 ▸).

**Figure 5:**
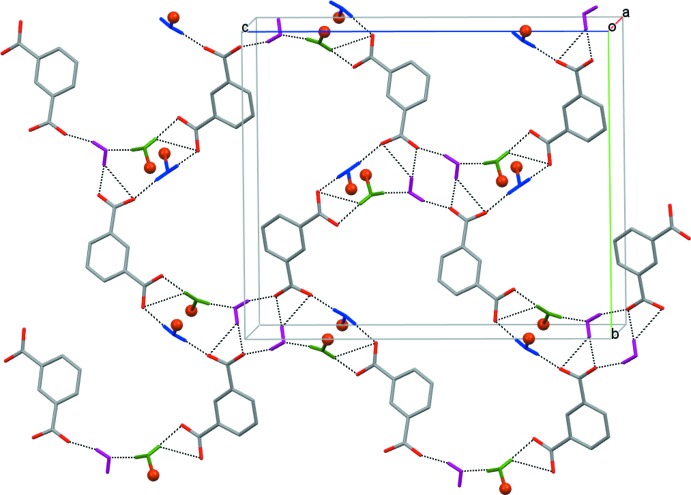
Sheets of isophthalate dianions parallel to the (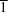
01) plane in (I). Macrocyclic ligands and H atoms at carbon atoms of the carboxyl­ate anions are omitted, only water mol­ecules coordinated to Cu^II^ (balls) participating in the formation of a carboxyl­ate layer are shown (O1*W* – green, O2*W* – dark blue, O3*W* – violet). Hydrogen bonds are shown as dashed lines.

As can be seen from Figs. 3 ▸ and 4 ▸, because of the hydrogen bonding, two pairs of isophthalate anions are situated above and below the imaginary plane of the macrocyclic ligand. Each pair is further bound with symmetry-related partners *via* hydrogen bonding with the water mol­ecule of crystallization, O3*W*, thus forming layers of anions lying parallel to the (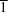
01) and (100) planes in (I) and (II), respectively (Figs. 5 ▸ and 6 ▸), which thus are pillared with macrocyclic cations.

## Database survey   

A search of the Cambridge Structural Database (CSD, version 5.39, last update August 2018; Groom *et al.*, 2016 ▸) indicated that only three Cu^II^–perchlorate complexes of aza­cyclam macrocycles bearing *N*-alkyl groups decorated with aromatic rings have been reported (Tsymbal *et al.*, 2010 ▸). In addition, four related dicopper(II) complexes with a *p*-xylylene-bridged bis­(aza­cyclam) ligand and terephthalate anion have been described, none of which includes the di­aqua Cu^II^ aza­cyclam cation (Park & Suh, 2012 ▸). At the same time, four complexes containing macrocyclic cations and an isophthalate dianion have been reported, all of them being formed by an Ni^II^ ion coordinated to a C-methyl-substituted cyclam. Thus, the title compounds (I) and (II) are the first examples of di­aqua Cu^II^ aza­cyclam cations described so far.

## Synthesis and crystallization   

All chemicals and solvents used in this work were purchased from Sigma–Aldrich and used without further purification. The starting complexes, [Cu(*L1*)](ClO_4_)_2_ and [Cu(*L2*)](ClO_4_)_2_, were prepared by a method reported in the literature (Tsymbal *et al.*, 2010 ▸) using benzyl­amine or 3-picolyl­amine, respectively, as locking reagents.

Compound (I) was prepared as follows: To a hot solution of [Cu(*L1*)](ClO_4_)_2_ (138 mg, 0.25 mmol) in 8 ml of DMF were added 3 ml of an aqueous solution of Na_2_ip (84 mg, 40 mmol). A violet precipitate formed in 24 h; this was filtered off, washed with diethyl ether and dried in air. Yield: 27 mg (19%). Analysis calculated for C_24_H_39_N_5_CuO_7_: C 50.29, H 6.86, N 12.22%. Found: C 50.42, H 6.96, N 12.02%.

Compound (II) was prepared analogously starting from [Cu(*L2*)](ClO_4_)_2_. Yield: 30 mg (21%). Analysis calculated for C_23_H_37.8_N_6_CuO_6.9_: C 48.12, H 6.67, N 14.64%. Found: C 48.31, H 6.84, N 14.32%. Violet plates of (I) and violet needles of (II) suitable for X-ray diffraction analysis were selected from the samples resulting from the syntheses.

**Safety note**: perchlorate salts of metal complexes are potentially explosive and should be handled with care.

## Refinement   

Crystal data, data collection and structure refinement details are summarized in Table 4 ▸. All H atoms in (I) were placed in geometrically idealized positions and constrained to ride on their parent atoms, with C—H distances of 0.93 (ring H atoms) or 0.97 Å (open-chain H atoms), an N—H distance of 0.98 Å, and aqua O—H distances of 0.84–0.87 Å with *U*_iso_(H) values of 1.2 or 1.5*U*_eq_ of the parent atoms. Water H atoms in (II) were positioned geometrically (O—H = 0.71–0.85 Å) and refined as riding with *U*_iso_(H) = 1.5*U*_eq_(O). All other H atoms were freely refined.

## Supplementary Material

Crystal structure: contains datablock(s) I, II. DOI: 10.1107/S2056989019008387/hb7828sup1.cif

Structure factors: contains datablock(s) I. DOI: 10.1107/S2056989019008387/hb7828Isup2.hkl

Structure factors: contains datablock(s) II. DOI: 10.1107/S2056989019008387/hb7828IIsup3.hkl

CCDC references: 1922733, 1922732

Additional supporting information:  crystallographic information; 3D view; checkCIF report

## Figures and Tables

**Figure 6:**
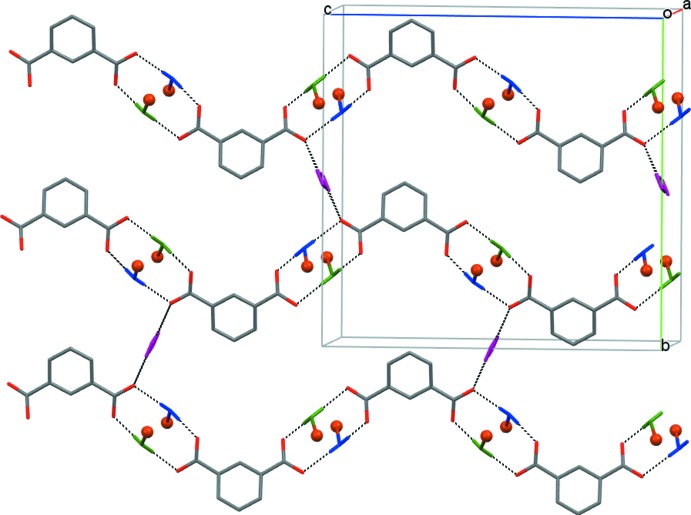
Sheets of isophthalate dianions parallel to the (100) plane in (II). Macrocyclic ligands and H atoms at carbon atoms of the carboxyl­ate anions are omitted, only water mol­ecules coordinated to Cu^II^ (balls) participating in the formation of a carboxyl­ate layer are shown (O1*W* – green, O2*W* – dark blue, O3*W* – violet). Hydrogen bonds are shown as dashed lines.

**Table 1:** Selected bond lengths (Å)

	(I)	(II)
Cu1—N1	2.0146 (17)	2.011 (3)
Cu1—N2	2.0290 (17)	2.019 (3)
Cu1—N4	2.0119 (17)	2.019 (3)
Cu1—N5	2.0206 (17)	2.009 (3)
Cu1—O1*W*	2.5071 (16)	2.514 (2)
Cu1—O2*W*	2.4832 (15)	2.499 (2)

**Table 2:** Hydrogen-bond geometry (Å, °) for (I)

*D*—H⋯*A*	*D*—H	H⋯*A*	*D*⋯*A*	*D*—H⋯*A*
N1—H1⋯O4^i^	0.98	2.04	2.950 (2)	154
N2—H2⋯O3^ii^	0.98	2.15	3.118 (2)	170
N4—H4⋯O2^iii^	0.98	2.00	2.949 (2)	161
N5—H5⋯O3*W* ^iv^	0.98	2.35	3.230 (3)	149
O1*W*—H1*WB*⋯O4^ii^	0.87	2.01	2.884 (2)	176
O1*W*—H1*WB*⋯O3^ii^	0.87	2.60	3.213 (2)	128
O1*W*—H1*WA*⋯O3*W* ^iii^	0.85	1.97	2.813 (2)	173
O2*W*—H2*WA*⋯O2^iv^	0.84	1.96	2.795 (2)	174
O2*W*—H2*WB*⋯O3^i^	0.85	1.95	2.798 (2)	178
O3*W*—H3*WA*⋯O1^v^	0.88	1.92	2.779 (2)	163
O3*W*—H3*WB*⋯O1	0.87	1.85	2.720 (2)	176
O3*W*—H3*WB*⋯O2	0.87	2.66	3.248 (2)	126

Symmetry codes: (i) 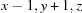; (ii) 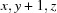; (iii) 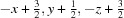; (iv) 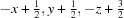; (v) 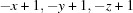.

**Table 3:** Hydrogen-bond geometry (Å, °) for (II)

*D*—H⋯*A*	*D*—H	H⋯*A*	*D*⋯*A*	*D*—H⋯*A*
N1—H1⋯O4	0.79 (4)	2.37 (4)	3.115 (4)	157 (4)
N2—H2⋯O3^i^	0.81 (3)	2.25 (4)	3.044 (4)	167 (3)
N4—H4⋯O1^ii^	0.78 (3)	2.31 (3)	3.037 (4)	157 (3)
N5—H5⋯O2^iii^	0.83 (4)	2.10 (4)	2.910 (4)	163 (3)
O1*W*—H1*WA*⋯O4^i^	0.85	2.00	2.849 (3)	174
O1*W*—H1*WB*⋯O2^ii^	0.76	2.07	2.831 (3)	176
O2*W*—H2*WA*⋯O1^iii^	0.71	2.15	2.859 (3)	178
O2*W*—H2*WB*⋯O3	0.82	1.90	2.722 (3)	180
O3*W*—H3*WA*⋯O1^ii^	0.85	1.88	2.731 (6)	179
O3*W*—H3*WB*⋯O1^iv^	0.85	2.18	2.760 (6)	126
C1—H1*A*⋯O4^i^	1.05 (4)	2.64 (4)	3.662 (5)	164 (3)
C4—H4*B*⋯O3*W* ^v^	0.98 (4)	2.60 (4)	3.274 (7)	125 (3)
C5—H5*B*⋯O3*W* ^v^	0.94 (4)	2.62 (4)	3.415 (7)	142 (3)
C10—H10*A*⋯O3*W*	0.92 (4)	2.49 (4)	3.367 (8)	161 (3)
C13—H13⋯O2^i^	1.03 (4)	2.53 (4)	3.446 (6)	147 (3)
C1—H1*B*⋯N6^vi^	0.84 (4)	2.66 (4)	3.474 (5)	165 (4)

Symmetry codes: (i) 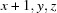; (ii) 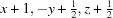; (iii) 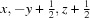; (iv) 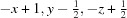; (v) 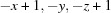; (vi) 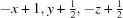.

**Table 4:** Experimental details

	(I)	(II)
Crystal data		
Chemical formula	[Cu(C_16_H_29_N_5_)(H_2_O)_2_](C_8_H_4_O_4_)·H_2_O	[Cu(C_15_H_28_N_6_)(H_2_O)_2_](C_8_H_4_O_4_)·0.9H_2_O
*M*_r_	573.14	572.33
Crystal system, space group	Monoclinic, *P*2_1_/*n*	Monoclinic, *P*2_1_/*c*
Temperature (K)	296	296
*a*, *b*, *c* (Å)	7.2625 (3), 17.8132 (7), 21.1511 (9)	7.1955 (3), 19.0463 (8), 19.4426 (8)
β (°)	92.159 (3)	94.276 (2)
*V* (Å^3^)	2734.34 (19)	2657.15 (19)
*Z*	4	4
Radiation type	Mo *K*α	Mo *K*α
μ (mm^−1^)	0.85	0.88
Crystal size (mm)	0.30 × 0.25 × 0.04	0.16 × 0.04 × 0.04

Data cocollection		
Diffractometer	Bruker X8 APEXII CCD	Bruker X8 APEXII CCD
Absorption correction	Multi-scan (*SADABS*; Bruker, 2007 ▸)	Multi-scan (*SADABS*; Bruker, 2007 ▸)
*T*_min_, *T*_max_	0.785, 0.967	0.873, 0.966
No. of measured, independent and observed [*I* > 2σ(*I*)] reflections	137978, 5555, 4193	76082, 4532, 2834
*R*_int_	0.070	0.106
(sin θ/λ)_max_ (Å^−1^)	0.624	0.589

Refinement		
*R*[*F* ^2^ > 2σ(*F* ^2^)], *wR*(*F* ^2^), *S*	0.032, 0.084, 1.02	0.040, 0.097, 1.00
No. of reflections	5555	4532
No. of parameters	334	439
No. of restraints	9	0
H-atom treatment	H-atom parameters constrained	H atoms treated by a mixture of independent and constrained refinement
Δρ_max_, Δρ_min_ (e Å^−3^)	0.25, −0.24	0.29, −0.34

Computer programs: *APEX2* and *SAINT* (Bruker, 2007 ▸), *SHELXS2014* (Sheldrick, 2015*a* ▸), *SHELXL2014* (Sheldrick, 2015*b* ▸), *Mercury* (Macrae *et al.*, 2008 ▸) and *publCIF* (Westrip, 2010 ▸).

## supplementary crystallographic information

### trans-Diaqua(3-benzyl-1,3,5,8,12-pentaazacyclotetradecane-κ4N1,N5,N8,N12)copper(II) isophthalate monohydrate (I) Crystal data

**Table:**

[Cu(C_16_H_29_N_5_)(H_2_O)_2_](C_8_H_4_O_4_)·H_2_O	*F*(000) = 1212
*M**_r_* = 573.14	*D*_x_ = 1.392 Mg m^−^^3^
Monoclinic, *P*2_1_/*n*	Mo *K*α radiation, λ = 0.71073 Å
*a* = 7.2625 (3) Å	Cell parameters from 6434 reflections
*b* = 17.8132 (7) Å	θ = 2.9–24.8°
*c* = 21.1511 (9) Å	µ = 0.85 mm^−^^1^
β = 92.159 (3)°	*T* = 296 K
*V* = 2734.34 (19) Å^3^	Plate, violet
*Z* = 4	0.30 × 0.25 × 0.04 mm

### trans-Diaqua(3-benzyl-1,3,5,8,12-pentaazacyclotetradecane-κ4N1,N5,N8,N12)copper(II) isophthalate monohydrate (I) Data collection

**Table:**

Bruker X8 APEXII CCD diffractometer	4193 reflections with *I* > 2σ(*I*)
Radiation source: fine-focus sealed tube	*R*_int_ = 0.070
φ and ω scans	θ_max_ = 26.3°, θ_min_ = 2.5°
Absorption correction: multi-scan (SADABS; Bruker, 2007)	*h* = −9→9
*T*_min_ = 0.785, *T*_max_ = 0.967	*k* = −22→22
137978 measured reflections	*l* = −26→26
5555 independent reflections	

### trans-Diaqua(3-benzyl-1,3,5,8,12-pentaazacyclotetradecane-κ4N1,N5,N8,N12)copper(II) isophthalate monohydrate (I) Refinement

**Table:**

Refinement on *F*^2^	Primary atom site location: structure-invariant direct methods
Least-squares matrix: full	Hydrogen site location: mixed
*R*[*F*^2^ > 2σ(*F*^2^)] = 0.032	H-atom parameters constrained
*wR*(*F*^2^) = 0.084	*w* = 1/[σ^2^(*F*_o_^2^) + (0.0366*P*)^2^ + 1.2028*P*] where *P* = (*F*_o_^2^ + 2*F*_c_^2^)/3
*S* = 1.02	(Δ/σ)_max_ = 0.001
5555 reflections	Δρ_max_ = 0.25 e Å^−^^3^
334 parameters	Δρ_min_ = −0.24 e Å^−^^3^
9 restraints	

### trans-Diaqua(3-benzyl-1,3,5,8,12-pentaazacyclotetradecane-κ4N1,N5,N8,N12)copper(II) isophthalate monohydrate (I) Special details

**Table:**

Geometry. All esds (except the esd in the dihedral angle between two l.s. planes) are estimated using the full covariance matrix. The cell esds are taken into account individually in the estimation of esds in distances, angles and torsion angles; correlations between esds in cell parameters are only used when they are defined by crystal symmetry. An approximate (isotropic) treatment of cell esds is used for estimating esds involving l.s. planes.

### trans-Diaqua(3-benzyl-1,3,5,8,12-pentaazacyclotetradecane-κ4N1,N5,N8,N12)copper(II) isophthalate monohydrate (I) Fractional atomic coordinates and isotropic or equivalent isotropic displacement parameters (Å^2^)

**Table:**

	*x*	*y*	*z*	*U*_iso_*/*U*_eq_	
Cu1	0.37585 (3)	1.01815 (2)	0.78932 (2)	0.03694 (9)	
O1	0.5450 (3)	0.40229 (9)	0.54740 (8)	0.0648 (5)	
O2	0.6548 (2)	0.39430 (8)	0.64621 (7)	0.0487 (4)	
O3	0.8446 (2)	0.05140 (8)	0.67653 (8)	0.0532 (4)	
O4	0.9001 (2)	0.15648 (9)	0.73008 (7)	0.0498 (4)	
O3W	0.5996 (3)	0.55120 (10)	0.56962 (8)	0.0648 (5)	
H3WA	0.5628	0.5751	0.5349	0.097*	
H3WB	0.5787	0.5037	0.5639	0.097*	
N1	0.2874 (2)	1.12200 (10)	0.76576 (8)	0.0401 (4)	
H1	0.1526	1.1199	0.7621	0.048*	
N2	0.4363 (2)	1.00637 (9)	0.69696 (8)	0.0375 (4)	
H2	0.5687	1.0154	0.6936	0.045*	
N3	0.4758 (2)	0.86889 (10)	0.70197 (9)	0.0429 (4)	
N4	0.4561 (2)	0.91310 (9)	0.81190 (8)	0.0374 (4)	
H4	0.5906	0.9132	0.8175	0.045*	
N5	0.3123 (2)	1.02892 (11)	0.88106 (8)	0.0432 (4)	
H5	0.1787	1.0222	0.8828	0.052*	
C1	0.3531 (3)	1.13819 (13)	0.70214 (11)	0.0502 (6)	
H1A	0.4803	1.1548	0.7051	0.060*	
H1B	0.2794	1.1778	0.6825	0.060*	
C2	0.3377 (3)	1.06781 (13)	0.66276 (10)	0.0478 (6)	
H2A	0.2091	1.0545	0.6555	0.057*	
H2B	0.3916	1.0760	0.6221	0.057*	
C3	0.3946 (3)	0.93131 (12)	0.66827 (10)	0.0441 (5)	
H3A	0.2620	0.9245	0.6658	0.053*	
H3B	0.4374	0.9309	0.6254	0.053*	
C4	0.4046 (3)	0.85523 (12)	0.76317 (11)	0.0446 (5)	
H4A	0.4487	0.8067	0.7781	0.054*	
H4B	0.2713	0.8525	0.7590	0.054*	
C5	0.3781 (3)	0.89603 (13)	0.87395 (10)	0.0473 (6)	
H5A	0.4432	0.8541	0.8936	0.057*	
H5B	0.2491	0.8826	0.8684	0.057*	
C6	0.3982 (3)	0.96466 (14)	0.91516 (10)	0.0478 (6)	
H6A	0.3382	0.9565	0.9548	0.057*	
H6B	0.5276	0.9749	0.9245	0.057*	
C7	0.3557 (4)	1.10175 (14)	0.91163 (11)	0.0547 (6)	
H7A	0.4882	1.1091	0.9133	0.066*	
H7B	0.3146	1.1009	0.9547	0.066*	
C8	0.2646 (4)	1.16677 (15)	0.87651 (12)	0.0619 (7)	
H8A	0.1334	1.1569	0.8722	0.074*	
H8B	0.2807	1.2117	0.9020	0.074*	
C9	0.3361 (3)	1.18222 (13)	0.81163 (12)	0.0533 (6)	
H9A	0.2854	1.2294	0.7961	0.064*	
H9B	0.4691	1.1873	0.8149	0.064*	
C10	0.6775 (3)	0.86203 (15)	0.69872 (11)	0.0517 (6)	
H10A	0.7207	0.8244	0.7290	0.062*	
H10B	0.7337	0.9095	0.7109	0.062*	
C11	0.7397 (3)	0.84076 (13)	0.63423 (12)	0.0471 (6)	
C12	0.7251 (3)	0.76725 (14)	0.61308 (14)	0.0579 (7)	
H12	0.6735	0.7310	0.6387	0.069*	
C13	0.7863 (4)	0.74730 (17)	0.55453 (16)	0.0745 (9)	
H13	0.7773	0.6977	0.5412	0.089*	
C14	0.8599 (5)	0.7999 (2)	0.51605 (17)	0.0875 (10)	
H14	0.9000	0.7864	0.4764	0.105*	
C15	0.8740 (5)	0.8718 (2)	0.53593 (17)	0.0938 (11)	
H15	0.9238	0.9077	0.5097	0.113*	
C16	0.8155 (4)	0.89255 (16)	0.59478 (14)	0.0696 (8)	
H16	0.8276	0.9422	0.6079	0.084*	
C17	0.6606 (3)	0.28470 (11)	0.58309 (9)	0.0336 (4)	
C18	0.6232 (3)	0.25013 (13)	0.52558 (10)	0.0467 (6)	
H18	0.5736	0.2779	0.4918	0.056*	
C19	0.6587 (4)	0.17463 (14)	0.51781 (11)	0.0573 (7)	
H19	0.6332	0.1519	0.4789	0.069*	
C20	0.7320 (3)	0.13288 (12)	0.56760 (10)	0.0473 (5)	
H20	0.7542	0.0819	0.5623	0.057*	
C21	0.7726 (3)	0.16653 (11)	0.62556 (9)	0.0333 (4)	
C22	0.7357 (3)	0.24223 (11)	0.63274 (9)	0.0321 (4)	
H22	0.7617	0.2651	0.6716	0.039*	
C23	0.6174 (3)	0.36688 (12)	0.59299 (10)	0.0391 (5)	
C24	0.8464 (3)	0.12152 (12)	0.68160 (10)	0.0372 (5)	
O2W	0.05598 (19)	0.97303 (9)	0.76801 (8)	0.0524 (4)	
H2WA	−0.0020	0.9507	0.7957	0.079*	
H2WB	−0.0090	0.9958	0.7401	0.079*	
O1W	0.6919 (2)	1.06796 (10)	0.81644 (7)	0.0537 (4)	
H1WA	0.7519	1.0591	0.8508	0.081*	
H1WB	0.7511	1.0939	0.7887	0.081*	

### trans-Diaqua(3-benzyl-1,3,5,8,12-pentaazacyclotetradecane-κ4N1,N5,N8,N12)copper(II) isophthalate monohydrate (I) Atomic displacement parameters (Å^2^)

**Table:**

	*U*^11^	*U*^22^	*U*^33^	*U*^12^	*U*^13^	*U*^23^
Cu1	0.03746 (15)	0.03986 (14)	0.03343 (14)	0.00188 (11)	0.00046 (10)	0.00340 (11)
O1	0.0916 (14)	0.0464 (9)	0.0544 (10)	0.0095 (9)	−0.0229 (10)	0.0123 (8)
O2	0.0523 (10)	0.0434 (8)	0.0498 (9)	0.0096 (7)	−0.0069 (8)	−0.0060 (7)
O3	0.0622 (11)	0.0351 (8)	0.0616 (10)	0.0028 (7)	−0.0075 (8)	0.0095 (8)
O4	0.0569 (10)	0.0465 (9)	0.0449 (9)	0.0043 (7)	−0.0150 (8)	0.0056 (7)
O3W	0.0889 (14)	0.0498 (10)	0.0538 (10)	0.0027 (9)	−0.0241 (10)	0.0014 (8)
N1	0.0305 (9)	0.0447 (10)	0.0450 (10)	0.0021 (8)	0.0004 (8)	0.0029 (8)
N2	0.0298 (9)	0.0452 (10)	0.0374 (9)	0.0005 (7)	0.0000 (7)	0.0046 (8)
N3	0.0361 (10)	0.0463 (10)	0.0461 (10)	0.0027 (8)	−0.0019 (8)	−0.0040 (8)
N4	0.0302 (9)	0.0430 (10)	0.0388 (9)	0.0005 (7)	−0.0001 (7)	0.0053 (8)
N5	0.0323 (10)	0.0586 (12)	0.0386 (10)	0.0044 (8)	0.0018 (8)	0.0006 (9)
C1	0.0470 (14)	0.0473 (13)	0.0569 (15)	0.0057 (11)	0.0096 (11)	0.0173 (11)
C2	0.0450 (13)	0.0606 (15)	0.0380 (12)	0.0058 (11)	0.0027 (10)	0.0151 (11)
C3	0.0384 (12)	0.0541 (13)	0.0394 (12)	−0.0003 (10)	−0.0054 (10)	−0.0062 (10)
C4	0.0380 (12)	0.0415 (12)	0.0542 (14)	−0.0048 (10)	0.0008 (10)	0.0006 (10)
C5	0.0426 (13)	0.0539 (14)	0.0455 (13)	0.0003 (11)	0.0051 (10)	0.0159 (11)
C6	0.0404 (13)	0.0688 (16)	0.0343 (11)	0.0036 (11)	0.0019 (10)	0.0103 (11)
C7	0.0524 (15)	0.0682 (16)	0.0436 (13)	0.0079 (12)	0.0010 (11)	−0.0132 (12)
C8	0.0598 (17)	0.0633 (16)	0.0624 (16)	0.0155 (13)	0.0003 (13)	−0.0174 (13)
C9	0.0513 (15)	0.0437 (13)	0.0643 (16)	0.0066 (11)	−0.0051 (12)	−0.0030 (12)
C10	0.0400 (13)	0.0639 (15)	0.0507 (14)	0.0025 (11)	−0.0046 (11)	−0.0016 (12)
C11	0.0353 (12)	0.0454 (13)	0.0602 (15)	0.0056 (10)	−0.0024 (11)	−0.0046 (11)
C12	0.0420 (14)	0.0463 (13)	0.0846 (19)	0.0036 (11)	−0.0067 (13)	−0.0028 (13)
C13	0.0559 (18)	0.0638 (18)	0.103 (2)	0.0145 (14)	−0.0066 (17)	−0.0377 (18)
C14	0.077 (2)	0.103 (3)	0.083 (2)	0.005 (2)	0.0206 (18)	−0.037 (2)
C15	0.108 (3)	0.088 (2)	0.088 (2)	−0.008 (2)	0.044 (2)	−0.007 (2)
C16	0.079 (2)	0.0523 (15)	0.079 (2)	−0.0034 (14)	0.0246 (16)	−0.0112 (14)
C17	0.0288 (10)	0.0382 (11)	0.0336 (10)	−0.0040 (8)	−0.0005 (8)	0.0051 (8)
C18	0.0556 (15)	0.0505 (13)	0.0333 (11)	−0.0019 (11)	−0.0086 (10)	0.0068 (10)
C19	0.0835 (19)	0.0545 (14)	0.0330 (12)	−0.0011 (13)	−0.0084 (12)	−0.0083 (11)
C20	0.0591 (15)	0.0392 (12)	0.0437 (13)	0.0008 (11)	0.0013 (11)	−0.0041 (10)
C21	0.0292 (11)	0.0360 (10)	0.0349 (10)	−0.0020 (8)	0.0025 (8)	0.0041 (8)
C22	0.0288 (10)	0.0375 (10)	0.0299 (10)	−0.0036 (8)	−0.0005 (8)	0.0007 (8)
C23	0.0341 (12)	0.0401 (11)	0.0430 (12)	−0.0015 (9)	−0.0011 (9)	0.0060 (10)
C24	0.0277 (11)	0.0413 (12)	0.0428 (12)	0.0004 (9)	0.0026 (9)	0.0069 (10)
O2W	0.0305 (8)	0.0620 (10)	0.0642 (10)	−0.0010 (7)	−0.0034 (7)	0.0196 (8)
O1W	0.0393 (9)	0.0735 (11)	0.0481 (9)	−0.0017 (8)	−0.0029 (7)	0.0129 (8)

### trans-Diaqua(3-benzyl-1,3,5,8,12-pentaazacyclotetradecane-κ4N1,N5,N8,N12)copper(II) isophthalate monohydrate (I) Geometric parameters (Å, º)

**Table:**

Cu1—N4	2.0119 (17)	C7—C8	1.515 (3)
Cu1—N1	2.0146 (17)	C7—H7A	0.9700
Cu1—N5	2.0206 (17)	C7—H7B	0.9700
Cu1—N2	2.0290 (17)	C8—C9	1.511 (4)
O1—C23	1.251 (2)	C8—H8A	0.9700
O2—C23	1.247 (2)	C8—H8B	0.9700
O3—C24	1.254 (3)	C9—H9A	0.9700
O4—C24	1.249 (3)	C9—H9B	0.9700
O3W—H3WA	0.8814	C10—C11	1.502 (3)
O3W—H3WB	0.8671	C10—H10A	0.9700
N1—C1	1.473 (3)	C10—H10B	0.9700
N1—C9	1.480 (3)	C11—C16	1.373 (4)
N1—H1	0.9800	C11—C12	1.386 (3)
N2—C2	1.481 (3)	C12—C13	1.378 (4)
N2—C3	1.495 (3)	C12—H12	0.9300
N2—H2	0.9800	C13—C14	1.364 (5)
N3—C4	1.433 (3)	C13—H13	0.9300
N3—C3	1.435 (3)	C14—C15	1.350 (5)
N3—C10	1.474 (3)	C14—H14	0.9300
N4—C5	1.480 (3)	C15—C16	1.381 (4)
N4—C4	1.495 (3)	C15—H15	0.9300
N4—H4	0.9800	C16—H16	0.9300
N5—C6	1.478 (3)	C17—C18	1.381 (3)
N5—C7	1.478 (3)	C17—C22	1.389 (3)
N5—H5	0.9800	C17—C23	1.513 (3)
C1—C2	1.507 (3)	C18—C19	1.380 (3)
C1—H1A	0.9700	C18—H18	0.9300
C1—H1B	0.9700	C19—C20	1.380 (3)
C2—H2A	0.9700	C19—H19	0.9300
C2—H2B	0.9700	C20—C21	1.386 (3)
C3—H3A	0.9700	C20—H20	0.9300
C3—H3B	0.9700	C21—C22	1.384 (3)
C4—H4A	0.9700	C21—C24	1.513 (3)
C4—H4B	0.9700	C22—H22	0.9300
C5—C6	1.505 (3)	O2W—H2WA	0.8360
C5—H5A	0.9700	O2W—H2WB	0.8450
C5—H5B	0.9700	O1W—H1WA	0.8473
C6—H6A	0.9700	O1W—H1WB	0.8730
C6—H6B	0.9700		
			
N4—Cu1—N1	178.13 (7)	H6A—C6—H6B	108.4
N4—Cu1—N5	86.27 (7)	N5—C7—C8	112.00 (19)
N1—Cu1—N5	93.91 (7)	N5—C7—H7A	109.2
N4—Cu1—N2	93.51 (7)	C8—C7—H7A	109.2
N1—Cu1—N2	86.29 (7)	N5—C7—H7B	109.2
N5—Cu1—N2	179.15 (8)	C8—C7—H7B	109.2
H3WA—O3W—H3WB	108.0	H7A—C7—H7B	107.9
C1—N1—C9	112.30 (18)	C9—C8—C7	115.2 (2)
C1—N1—Cu1	107.13 (13)	C9—C8—H8A	108.5
C9—N1—Cu1	115.94 (13)	C7—C8—H8A	108.5
C1—N1—H1	107.0	C9—C8—H8B	108.5
C9—N1—H1	107.0	C7—C8—H8B	108.5
Cu1—N1—H1	107.0	H8A—C8—H8B	107.5
C2—N2—C3	112.08 (17)	N1—C9—C8	112.4 (2)
C2—N2—Cu1	106.01 (13)	N1—C9—H9A	109.1
C3—N2—Cu1	115.82 (13)	C8—C9—H9A	109.1
C2—N2—H2	107.5	N1—C9—H9B	109.1
C3—N2—H2	107.5	C8—C9—H9B	109.1
Cu1—N2—H2	107.5	H9A—C9—H9B	107.8
C4—N3—C3	115.14 (18)	N3—C10—C11	113.38 (18)
C4—N3—C10	114.89 (18)	N3—C10—H10A	108.9
C3—N3—C10	115.56 (19)	C11—C10—H10A	108.9
C5—N4—C4	112.06 (17)	N3—C10—H10B	108.9
C5—N4—Cu1	106.53 (13)	C11—C10—H10B	108.9
C4—N4—Cu1	114.52 (13)	H10A—C10—H10B	107.7
C5—N4—H4	107.8	C16—C11—C12	117.8 (2)
C4—N4—H4	107.8	C16—C11—C10	121.6 (2)
Cu1—N4—H4	107.8	C12—C11—C10	120.6 (2)
C6—N5—C7	112.77 (18)	C13—C12—C11	120.7 (3)
C6—N5—Cu1	106.72 (13)	C13—C12—H12	119.7
C7—N5—Cu1	116.79 (15)	C11—C12—H12	119.7
C6—N5—H5	106.7	C14—C13—C12	120.4 (3)
C7—N5—H5	106.7	C14—C13—H13	119.8
Cu1—N5—H5	106.7	C12—C13—H13	119.8
N1—C1—C2	108.80 (18)	C15—C14—C13	119.5 (3)
N1—C1—H1A	109.9	C15—C14—H14	120.2
C2—C1—H1A	109.9	C13—C14—H14	120.2
N1—C1—H1B	109.9	C14—C15—C16	120.8 (3)
C2—C1—H1B	109.9	C14—C15—H15	119.6
H1A—C1—H1B	108.3	C16—C15—H15	119.6
N2—C2—C1	108.68 (18)	C11—C16—C15	120.8 (3)
N2—C2—H2A	110.0	C11—C16—H16	119.6
C1—C2—H2A	110.0	C15—C16—H16	119.6
N2—C2—H2B	110.0	C18—C17—C22	118.79 (19)
C1—C2—H2B	110.0	C18—C17—C23	121.21 (18)
H2A—C2—H2B	108.3	C22—C17—C23	119.98 (18)
N3—C3—N2	114.72 (17)	C19—C18—C17	120.5 (2)
N3—C3—H3A	108.6	C19—C18—H18	119.7
N2—C3—H3A	108.6	C17—C18—H18	119.7
N3—C3—H3B	108.6	C20—C19—C18	120.2 (2)
N2—C3—H3B	108.6	C20—C19—H19	119.9
H3A—C3—H3B	107.6	C18—C19—H19	119.9
N3—C4—N4	114.61 (17)	C19—C20—C21	120.3 (2)
N3—C4—H4A	108.6	C19—C20—H20	119.8
N4—C4—H4A	108.6	C21—C20—H20	119.8
N3—C4—H4B	108.6	C22—C21—C20	118.86 (19)
N4—C4—H4B	108.6	C22—C21—C24	119.66 (18)
H4A—C4—H4B	107.6	C20—C21—C24	121.41 (18)
N4—C5—C6	108.34 (18)	C21—C22—C17	121.31 (18)
N4—C5—H5A	110.0	C21—C22—H22	119.3
C6—C5—H5A	110.0	C17—C22—H22	119.3
N4—C5—H5B	110.0	O2—C23—O1	124.7 (2)
C6—C5—H5B	110.0	O2—C23—C17	117.66 (18)
H5A—C5—H5B	108.4	O1—C23—C17	117.66 (19)
N5—C6—C5	108.45 (17)	O4—C24—O3	124.7 (2)
N5—C6—H6A	110.0	O4—C24—C21	118.00 (18)
C5—C6—H6A	110.0	O3—C24—C21	117.30 (19)
N5—C6—H6B	110.0	H2WA—O2W—H2WB	115.8
C5—C6—H6B	110.0	H1WA—O1W—H1WB	115.1
			
C9—N1—C1—C2	167.12 (18)	N3—C10—C11—C12	−77.4 (3)
Cu1—N1—C1—C2	38.7 (2)	C16—C11—C12—C13	0.4 (4)
C3—N2—C2—C1	167.77 (18)	C10—C11—C12—C13	−178.4 (2)
Cu1—N2—C2—C1	40.5 (2)	C11—C12—C13—C14	−0.9 (4)
N1—C1—C2—N2	−54.0 (2)	C12—C13—C14—C15	0.6 (5)
C4—N3—C3—N2	67.2 (2)	C13—C14—C15—C16	0.2 (6)
C10—N3—C3—N2	−70.5 (2)	C12—C11—C16—C15	0.3 (4)
C2—N2—C3—N3	−175.81 (18)	C10—C11—C16—C15	179.2 (3)
Cu1—N2—C3—N3	−54.0 (2)	C14—C15—C16—C11	−0.7 (6)
C3—N3—C4—N4	−70.0 (2)	C22—C17—C18—C19	0.4 (3)
C10—N3—C4—N4	68.0 (2)	C23—C17—C18—C19	−178.2 (2)
C5—N4—C4—N3	179.82 (18)	C17—C18—C19—C20	0.1 (4)
Cu1—N4—C4—N3	58.3 (2)	C18—C19—C20—C21	−0.8 (4)
C4—N4—C5—C6	−166.99 (17)	C19—C20—C21—C22	1.0 (3)
Cu1—N4—C5—C6	−41.01 (19)	C19—C20—C21—C24	177.7 (2)
C7—N5—C6—C5	−168.77 (19)	C20—C21—C22—C17	−0.5 (3)
Cu1—N5—C6—C5	−39.2 (2)	C24—C21—C22—C17	−177.30 (18)
N4—C5—C6—N5	54.5 (2)	C18—C17—C22—C21	−0.2 (3)
C6—N5—C7—C8	−179.3 (2)	C23—C17—C22—C21	178.41 (18)
Cu1—N5—C7—C8	56.6 (2)	C18—C17—C23—O2	179.9 (2)
N5—C7—C8—C9	−67.4 (3)	C22—C17—C23—O2	1.3 (3)
C1—N1—C9—C8	178.50 (19)	C18—C17—C23—O1	0.6 (3)
Cu1—N1—C9—C8	−57.9 (2)	C22—C17—C23—O1	−178.0 (2)
C7—C8—C9—N1	68.5 (3)	C22—C21—C24—O4	−11.4 (3)
C4—N3—C10—C11	153.0 (2)	C20—C21—C24—O4	171.9 (2)
C3—N3—C10—C11	−69.1 (3)	C22—C21—C24—O3	166.69 (19)
N3—C10—C11—C16	103.8 (3)	C20—C21—C24—O3	−10.0 (3)

### trans-Diaqua(3-benzyl-1,3,5,8,12-pentaazacyclotetradecane-κ4N1,N5,N8,N12)copper(II) isophthalate monohydrate (I) Hydrogen-bond geometry (Å, º)

**Table:**

*D*—H···*A*	*D*—H	H···*A*	*D*···*A*	*D*—H···*A*
N1—H1···O4^i^	0.98	2.04	2.950 (2)	154
N2—H2···O3^ii^	0.98	2.15	3.118 (2)	170
N4—H4···O2^iii^	0.98	2.00	2.949 (2)	161
N5—H5···O3*W*^iv^	0.98	2.35	3.230 (3)	149
O1*W*—H1*WB*···O4^ii^	0.87	2.01	2.884 (2)	176
O1*W*—H1*WB*···O3^ii^	0.87	2.60	3.213 (2)	128
O1*W*—H1*WA*···O3*W*^iii^	0.85	1.97	2.813 (2)	173
O2*W*—H2*WA*···O2^iv^	0.84	1.96	2.795 (2)	174
O2*W*—H2*WB*···O3^i^	0.85	1.95	2.798 (2)	178
O3*W*—H3*WA*···O1^v^	0.88	1.92	2.779 (2)	163
O3*W*—H3*WB*···O1	0.87	1.85	2.720 (2)	176
O3*W*—H3*WB*···O2	0.87	2.66	3.248 (2)	126

Symmetry codes: (i) *x*−1, *y*+1, *z*; (ii) *x*, *y*+1, *z*; (iii) −*x*+3/2, *y*+1/2, −*z*+3/2; (iv) −*x*+1/2, *y*+1/2, −*z*+3/2; (v) −*x*+1, −*y*+1, −*z*+1.

### trans-Diaqua[3-(pyridin-3-ylmethyl)-1,3,5,8,12-pentaazacyclotetradecane-κ4N1,N5,N8,N12]copper(II) isophthalate 0.9-hydrate (II) Crystal data

**Table:**

[Cu(C_15_H_28_N_6_)(H_2_O)_2_](C_8_H_4_O_4_)·0.9H_2_O	*F*(000) = 1208
*M**_r_* = 572.33	*D*_x_ = 1.431 Mg m^−^^3^
Monoclinic, *P*2_1_/*c*	Mo *K*α radiation, λ = 0.71073 Å
*a* = 7.1955 (3) Å	Cell parameters from 6756 reflections
*b* = 19.0463 (8) Å	θ = 3.5–24.1°
*c* = 19.4426 (8) Å	µ = 0.88 mm^−^^1^
β = 94.276 (2)°	*T* = 296 K
*V* = 2657.15 (19) Å^3^	Needle, violet
*Z* = 4	0.16 × 0.04 × 0.04 mm

### trans-Diaqua[3-(pyridin-3-ylmethyl)-1,3,5,8,12-pentaazacyclotetradecane-κ4N1,N5,N8,N12]copper(II) isophthalate 0.9-hydrate (II) Data collection

**Table:**

Bruker X8 APEXII CCD diffractometer	2834 reflections with *I* > 2σ(*I*)
Radiation source: fine-focus sealed tube	*R*_int_ = 0.106
φ and ω scans	θ_max_ = 24.7°, θ_min_ = 2.4°
Absorption correction: multi-scan (SADABS; Bruker, 2007)	*h* = −8→8
*T*_min_ = 0.873, *T*_max_ = 0.966	*k* = −22→22
76082 measured reflections	*l* = −22→22
4532 independent reflections	

### trans-Diaqua[3-(pyridin-3-ylmethyl)-1,3,5,8,12-pentaazacyclotetradecane-κ4N1,N5,N8,N12]copper(II) isophthalate 0.9-hydrate (II) Refinement

**Table:**

Refinement on *F*^2^	Primary atom site location: structure-invariant direct methods
Least-squares matrix: full	Hydrogen site location: difference Fourier map
*R*[*F*^2^ > 2σ(*F*^2^)] = 0.040	H atoms treated by a mixture of independent and constrained refinement
*wR*(*F*^2^) = 0.097	*w* = 1/[σ^2^(*F*_o_^2^) + (0.0305*P*)^2^ + 2.3396*P*] where *P* = (*F*_o_^2^ + 2*F*_c_^2^)/3
*S* = 1.00	(Δ/σ)_max_ = 0.001
4532 reflections	Δρ_max_ = 0.29 e Å^−^^3^
439 parameters	Δρ_min_ = −0.34 e Å^−^^3^
0 restraints	

### trans-Diaqua[3-(pyridin-3-ylmethyl)-1,3,5,8,12-pentaazacyclotetradecane-κ4N1,N5,N8,N12]copper(II) isophthalate 0.9-hydrate (II) Special details

**Table:**

Geometry. All esds (except the esd in the dihedral angle between two l.s. planes) are estimated using the full covariance matrix. The cell esds are taken into account individually in the estimation of esds in distances, angles and torsion angles; correlations between esds in cell parameters are only used when they are defined by crystal symmetry. An approximate (isotropic) treatment of cell esds is used for estimating esds involving l.s. planes.

### trans-Diaqua[3-(pyridin-3-ylmethyl)-1,3,5,8,12-pentaazacyclotetradecane-κ4N1,N5,N8,N12]copper(II) isophthalate 0.9-hydrate (II) Fractional atomic coordinates and isotropic or equivalent isotropic displacement parameters (Å^2^)

**Table:**

	*x*	*y*	*z*	*U*_iso_*/*U*_eq_	Occ. (<1)
Cu1	0.43303 (5)	0.24763 (2)	0.48049 (2)	0.03543 (14)	
O1W	0.7535 (3)	0.29360 (13)	0.51575 (12)	0.0526 (7)	
H1WA	0.8135	0.3174	0.4879	0.079*	
H1WB	0.8207	0.2732	0.5405	0.079*	
O2W	0.1108 (3)	0.20190 (13)	0.45250 (12)	0.0489 (7)	
H2WA	0.0608	0.1826	0.4762	0.073*	
H2WB	0.0448	0.2250	0.4244	0.073*	
O2	−0.0118 (3)	0.28246 (13)	0.11247 (12)	0.0458 (6)	
O1	−0.0810 (4)	0.37745 (13)	0.05000 (12)	0.0514 (7)	
O3	−0.1053 (3)	0.27854 (13)	0.35932 (12)	0.0477 (6)	
O4	−0.0716 (4)	0.37804 (13)	0.41829 (12)	0.0520 (7)	
O3W	0.8843 (8)	−0.0087 (3)	0.4939 (3)	0.090 (2)	0.5
H3WA	0.8940	0.0323	0.5112	0.135*	0.5
H3WB	0.9961	−0.0217	0.4903	0.135*	0.5
N5	0.3655 (4)	0.25100 (17)	0.57886 (14)	0.0422 (7)	
H5	0.252 (5)	0.2428 (18)	0.5797 (17)	0.051*	
N4	0.5142 (4)	0.14778 (15)	0.50045 (15)	0.0380 (7)	
H4	0.622 (5)	0.1491 (18)	0.5042 (18)	0.046*	
N3	0.5197 (4)	0.11268 (15)	0.37866 (14)	0.0422 (7)	
N2	0.4917 (4)	0.24119 (15)	0.38076 (14)	0.0375 (7)	
H2	0.604 (5)	0.2455 (18)	0.3789 (17)	0.045*	
N1	0.3470 (4)	0.34611 (16)	0.45862 (17)	0.0459 (8)	
H1	0.237 (5)	0.342 (2)	0.4524 (19)	0.055*	
N6	0.7057 (5)	0.0207 (2)	0.1924 (2)	0.0773 (12)	
C6	0.4550 (6)	0.1900 (2)	0.6144 (2)	0.0517 (11)	
H6A	0.400 (5)	0.1804 (19)	0.658 (2)	0.062*	
H6B	0.579 (6)	0.2040 (19)	0.6278 (19)	0.062*	
C5	0.4414 (6)	0.1282 (2)	0.56692 (19)	0.0472 (10)	
H5A	0.511 (5)	0.0884 (19)	0.5869 (18)	0.057*	
H5B	0.317 (5)	0.1139 (18)	0.5564 (17)	0.057*	
C4	0.4585 (6)	0.0962 (2)	0.4450 (2)	0.0475 (10)	
H4A	0.504 (5)	0.052 (2)	0.4583 (18)	0.057*	
H4B	0.322 (5)	0.0956 (18)	0.4417 (17)	0.057*	
C3	0.4360 (5)	0.1735 (2)	0.34625 (19)	0.0454 (10)	
H3A	0.305 (5)	0.1701 (18)	0.3498 (17)	0.055*	
H3B	0.475 (5)	0.1762 (17)	0.2977 (18)	0.055*	
C2	0.4032 (6)	0.3025 (2)	0.3452 (2)	0.0558 (12)	
H2A	0.275 (6)	0.291 (2)	0.3319 (19)	0.067*	
H2B	0.461 (5)	0.3108 (19)	0.303 (2)	0.067*	
C1	0.4203 (6)	0.3651 (2)	0.3917 (2)	0.0594 (12)	
H1A	0.561 (6)	0.379 (2)	0.4029 (19)	0.071*	
H1B	0.370 (6)	0.402 (2)	0.375 (2)	0.071*	
C9	0.3921 (7)	0.3989 (2)	0.5132 (3)	0.0637 (13)	
H9A	0.349 (6)	0.441 (2)	0.496 (2)	0.076*	
H9B	0.529 (6)	0.405 (2)	0.522 (2)	0.076*	
C8	0.3075 (7)	0.3790 (3)	0.5796 (3)	0.0730 (15)	
H8A	0.321 (6)	0.414 (2)	0.609 (2)	0.088*	
H8B	0.167 (6)	0.372 (2)	0.575 (2)	0.088*	
C7	0.3989 (7)	0.3169 (3)	0.6173 (2)	0.0603 (12)	
H7A	0.527 (6)	0.323 (2)	0.625 (2)	0.072*	
H7B	0.352 (6)	0.313 (2)	0.664 (2)	0.072*	
C10	0.7173 (6)	0.1022 (2)	0.3693 (2)	0.0500 (10)	
H10A	0.756 (5)	0.064 (2)	0.3953 (19)	0.060*	
H10B	0.798 (5)	0.1392 (19)	0.3909 (18)	0.060*	
C11	0.7527 (5)	0.09148 (19)	0.29454 (19)	0.0433 (9)	
C15	0.6836 (6)	0.0338 (2)	0.2589 (3)	0.0634 (13)	
H15	0.622 (6)	0.003 (2)	0.280 (2)	0.076*	
C14	0.8046 (7)	0.0681 (3)	0.1601 (2)	0.0711 (14)	
H14	0.834 (6)	0.060 (2)	0.117 (2)	0.085*	
C13	0.8791 (6)	0.1269 (3)	0.1905 (2)	0.0639 (12)	
H13	0.957 (6)	0.161 (2)	0.163 (2)	0.077*	
C12	0.8528 (6)	0.1385 (2)	0.2583 (2)	0.0514 (10)	
H12	0.903 (5)	0.177 (2)	0.2834 (19)	0.062*	
C16	−0.0922 (4)	0.38525 (17)	0.17181 (16)	0.0315 (8)	
C17	−0.1431 (5)	0.45554 (19)	0.17099 (19)	0.0419 (9)	
H17	−0.154 (5)	0.4782 (18)	0.1319 (18)	0.050*	
C18	−0.1798 (5)	0.4895 (2)	0.2311 (2)	0.0484 (10)	
H18	−0.212 (5)	0.535 (2)	0.2291 (18)	0.058*	
C19	−0.1628 (5)	0.45471 (19)	0.2933 (2)	0.0423 (9)	
H19	−0.178 (5)	0.4782 (18)	0.3327 (18)	0.051*	
C20	−0.1130 (4)	0.38455 (17)	0.29563 (16)	0.0322 (8)	
C21	−0.0791 (4)	0.35064 (18)	0.23471 (18)	0.0333 (8)	
H21	−0.049 (4)	0.3043 (17)	0.2347 (16)	0.040*	
C22	−0.0577 (4)	0.3458 (2)	0.10677 (17)	0.0377 (8)	
C23	−0.0942 (4)	0.34456 (19)	0.36318 (18)	0.0366 (8)	
O4W	0.018 (3)	0.5169 (8)	0.4782 (9)	0.138 (5)	0.2
H4WA	−0.0066	0.4764	0.4615	0.207*	0.2
H4WB	0.0112	0.5119	0.5213	0.207*	0.2
O5W	0.086 (3)	0.5056 (8)	0.4928 (9)	0.138 (5)	0.2
H5WA	0.0741	0.4989	0.5361	0.207*	0.2
H5WB	0.0812	0.4718	0.4661	0.207*	0.2

### trans-Diaqua[3-(pyridin-3-ylmethyl)-1,3,5,8,12-pentaazacyclotetradecane-κ4N1,N5,N8,N12]copper(II) isophthalate 0.9-hydrate (II) Atomic displacement parameters (Å^2^)

**Table:**

	*U*^11^	*U*^22^	*U*^33^	*U*^12^	*U*^13^	*U*^23^
Cu1	0.0391 (2)	0.0350 (2)	0.0325 (2)	0.0007 (2)	0.00440 (15)	0.0006 (2)
O1W	0.0417 (15)	0.0661 (18)	0.0504 (16)	0.0018 (12)	0.0065 (12)	0.0141 (14)
O2W	0.0363 (14)	0.0672 (18)	0.0431 (15)	−0.0005 (12)	0.0028 (11)	0.0152 (13)
O2	0.0547 (16)	0.0430 (16)	0.0411 (15)	0.0053 (12)	0.0118 (12)	−0.0040 (12)
O1	0.0723 (18)	0.0507 (16)	0.0320 (15)	−0.0058 (13)	0.0091 (12)	0.0048 (13)
O3	0.0655 (17)	0.0388 (16)	0.0385 (15)	0.0013 (12)	0.0034 (12)	0.0051 (12)
O4	0.0690 (18)	0.0542 (17)	0.0323 (15)	−0.0054 (13)	0.0008 (12)	−0.0085 (13)
O3W	0.085 (4)	0.059 (4)	0.129 (6)	−0.009 (3)	0.025 (4)	−0.033 (4)
N5	0.0338 (15)	0.0535 (19)	0.0395 (17)	−0.0023 (17)	0.0042 (13)	−0.0067 (17)
N4	0.0367 (16)	0.0421 (18)	0.0349 (17)	0.0003 (14)	0.0015 (13)	0.0025 (14)
N3	0.0411 (18)	0.047 (2)	0.0386 (18)	0.0035 (14)	0.0065 (13)	−0.0064 (15)
N2	0.0330 (15)	0.0432 (19)	0.0360 (16)	0.0024 (15)	0.0016 (13)	0.0069 (15)
N1	0.0358 (17)	0.0402 (19)	0.063 (2)	0.0030 (15)	0.0099 (16)	0.0016 (16)
N6	0.066 (3)	0.086 (3)	0.079 (3)	−0.001 (2)	0.004 (2)	−0.048 (2)
C6	0.047 (2)	0.076 (3)	0.031 (2)	0.005 (2)	0.0010 (19)	0.006 (2)
C5	0.045 (2)	0.056 (3)	0.041 (2)	0.002 (2)	0.0042 (18)	0.018 (2)
C4	0.052 (2)	0.038 (2)	0.053 (3)	−0.0043 (19)	0.011 (2)	−0.004 (2)
C3	0.041 (2)	0.063 (3)	0.033 (2)	0.0004 (19)	0.0044 (17)	−0.009 (2)
C2	0.057 (3)	0.064 (3)	0.046 (3)	0.014 (2)	0.007 (2)	0.023 (2)
C1	0.055 (3)	0.046 (3)	0.079 (3)	0.011 (2)	0.016 (2)	0.024 (3)
C9	0.061 (3)	0.036 (2)	0.095 (4)	0.001 (2)	0.012 (3)	−0.014 (3)
C8	0.076 (3)	0.060 (3)	0.086 (4)	−0.001 (3)	0.027 (3)	−0.035 (3)
C7	0.059 (3)	0.071 (3)	0.053 (3)	−0.005 (2)	0.011 (2)	−0.025 (3)
C10	0.047 (2)	0.050 (3)	0.053 (3)	0.0033 (19)	0.0030 (19)	−0.006 (2)
C11	0.036 (2)	0.042 (2)	0.051 (2)	0.0065 (17)	0.0032 (17)	−0.0141 (19)
C15	0.053 (3)	0.061 (3)	0.078 (3)	−0.010 (2)	0.017 (2)	−0.022 (3)
C14	0.064 (3)	0.095 (4)	0.055 (3)	0.026 (3)	0.009 (3)	−0.015 (3)
C13	0.068 (3)	0.065 (3)	0.060 (3)	0.015 (2)	0.011 (2)	0.003 (3)
C12	0.049 (2)	0.041 (3)	0.064 (3)	0.0016 (19)	0.005 (2)	−0.006 (2)
C16	0.0283 (17)	0.034 (2)	0.032 (2)	−0.0023 (14)	0.0050 (14)	−0.0003 (16)
C17	0.048 (2)	0.041 (2)	0.037 (2)	0.0002 (17)	0.0023 (17)	0.0075 (19)
C18	0.063 (3)	0.029 (2)	0.053 (3)	0.0046 (19)	0.0011 (19)	0.002 (2)
C19	0.048 (2)	0.038 (2)	0.042 (2)	−0.0016 (17)	0.0083 (17)	−0.0067 (19)
C20	0.0277 (18)	0.035 (2)	0.034 (2)	−0.0026 (14)	0.0044 (14)	−0.0048 (16)
C21	0.0295 (18)	0.0323 (19)	0.038 (2)	0.0029 (15)	0.0023 (14)	−0.0015 (19)
C22	0.0327 (19)	0.047 (3)	0.034 (2)	−0.0061 (16)	0.0050 (15)	0.0005 (19)
C23	0.0298 (19)	0.044 (2)	0.037 (2)	−0.0023 (16)	0.0060 (15)	0.0016 (19)
O4W	0.16 (2)	0.121 (12)	0.135 (14)	−0.018 (11)	0.049 (10)	−0.014 (10)
O5W	0.16 (2)	0.121 (12)	0.135 (14)	−0.018 (11)	0.049 (10)	−0.014 (10)

### trans-Diaqua[3-(pyridin-3-ylmethyl)-1,3,5,8,12-pentaazacyclotetradecane-κ4N1,N5,N8,N12]copper(II) isophthalate 0.9-hydrate (II) Geometric parameters (Å, º)

**Table:**

Cu1—N5	2.009 (3)	C3—H3B	1.01 (3)
Cu1—N1	2.011 (3)	C2—C1	1.496 (6)
Cu1—N4	2.019 (3)	C2—H2A	0.96 (4)
Cu1—N2	2.019 (3)	C2—H2B	0.95 (4)
Cu1—O2W	2.499 (2)	C1—H1A	1.05 (4)
Cu1—O1W	2.514 (2)	C1—H1B	0.84 (4)
O1W—H1WA	0.8490	C9—C8	1.518 (7)
O1W—H1WB	0.7628	C9—H9A	0.91 (4)
O2W—H2WA	0.7075	C9—H9B	0.99 (4)
O2W—H2WB	0.8243	C8—C7	1.515 (7)
O2—C22	1.253 (4)	C8—H8A	0.88 (5)
O1—C22	1.258 (4)	C8—H8B	1.01 (5)
O3—C23	1.262 (4)	C7—H7A	0.93 (4)
O4—C23	1.247 (4)	C7—H7B	1.00 (4)
O3W—H3WA	0.8501	C10—C11	1.508 (5)
O3W—H3WB	0.8500	C10—H10A	0.92 (4)
N5—C7	1.472 (5)	C10—H10B	0.99 (4)
N5—C6	1.475 (5)	C11—C15	1.373 (5)
N5—H5	0.83 (4)	C11—C12	1.376 (5)
N4—C5	1.478 (4)	C15—H15	0.85 (4)
N4—C4	1.491 (5)	C14—C13	1.358 (7)
N4—H4	0.78 (3)	C14—H14	0.90 (4)
N3—C4	1.428 (5)	C13—C12	1.365 (6)
N3—C3	1.430 (5)	C13—H13	1.03 (4)
N3—C10	1.460 (5)	C12—H12	0.94 (4)
N2—C2	1.477 (5)	C16—C21	1.386 (4)
N2—C3	1.494 (5)	C16—C17	1.388 (5)
N2—H2	0.81 (3)	C16—C22	1.507 (4)
N1—C9	1.480 (5)	C17—C18	1.378 (5)
N1—C1	1.484 (5)	C17—H17	0.87 (3)
N1—H1	0.79 (4)	C18—C19	1.377 (5)
N6—C14	1.335 (6)	C18—H18	0.90 (4)
N6—C15	1.337 (6)	C19—C20	1.383 (5)
C6—C5	1.494 (6)	C19—H19	0.90 (3)
C6—H6A	0.98 (4)	C20—C21	1.387 (4)
C6—H6B	0.95 (4)	C20—C23	1.516 (4)
C5—H5A	0.97 (4)	C21—H21	0.91 (3)
C5—H5B	0.94 (4)	O4W—H4WA	0.8499
C4—H4A	0.94 (4)	O4W—H4WB	0.8499
C4—H4B	0.98 (4)	O5W—H5WA	0.8621
C3—H3A	0.95 (4)	O5W—H5WB	0.8273
			
N5—Cu1—N1	94.53 (13)	C1—C2—H2A	112 (2)
N5—Cu1—N4	86.23 (12)	N2—C2—H2B	109 (2)
N1—Cu1—N4	178.43 (13)	C1—C2—H2B	111 (2)
N5—Cu1—N2	177.48 (12)	H2A—C2—H2B	106 (3)
N1—Cu1—N2	86.37 (12)	N1—C1—C2	108.5 (3)
N4—Cu1—N2	92.82 (12)	N1—C1—H1A	106 (2)
N5—Cu1—O2W	86.10 (10)	C2—C1—H1A	111 (2)
N1—Cu1—O2W	90.74 (10)	N1—C1—H1B	111 (3)
N4—Cu1—O2W	87.94 (10)	C2—C1—H1B	115 (3)
N2—Cu1—O2W	91.54 (10)	H1A—C1—H1B	105 (4)
N5—Cu1—O1W	90.65 (10)	N1—C9—C8	111.0 (4)
N1—Cu1—O1W	89.69 (10)	N1—C9—H9A	106 (3)
N4—Cu1—O1W	91.68 (10)	C8—C9—H9A	113 (3)
N2—Cu1—O1W	91.71 (10)	N1—C9—H9B	111 (2)
O2W—Cu1—O1W	176.75 (8)	C8—C9—H9B	110 (2)
Cu1—O1W—H1WA	120.8	H9A—C9—H9B	106 (4)
Cu1—O1W—H1WB	121.4	C7—C8—C9	114.8 (4)
H1WA—O1W—H1WB	110.2	C7—C8—H8A	105 (3)
Cu1—O2W—H2WA	123.2	C9—C8—H8A	109 (3)
Cu1—O2W—H2WB	115.9	C7—C8—H8B	109 (3)
H2WA—O2W—H2WB	114.4	C9—C8—H8B	114 (2)
H3WA—O3W—H3WB	104.5	H8A—C8—H8B	103 (4)
C7—N5—C6	112.7 (3)	N5—C7—C8	111.8 (4)
C7—N5—Cu1	117.9 (3)	N5—C7—H7A	108 (2)
C6—N5—Cu1	107.0 (2)	C8—C7—H7A	111 (3)
C7—N5—H5	106 (2)	N5—C7—H7B	110 (2)
C6—N5—H5	104 (3)	C8—C7—H7B	110 (2)
Cu1—N5—H5	109 (2)	H7A—C7—H7B	106 (3)
C5—N4—C4	111.9 (3)	N3—C10—C11	112.0 (3)
C5—N4—Cu1	106.8 (2)	N3—C10—H10A	107 (2)
C4—N4—Cu1	115.0 (2)	C11—C10—H10A	111 (2)
C5—N4—H4	110 (3)	N3—C10—H10B	113 (2)
C4—N4—H4	108 (3)	C11—C10—H10B	112 (2)
Cu1—N4—H4	105 (3)	H10A—C10—H10B	101 (3)
C4—N3—C3	115.3 (3)	C15—C11—C12	116.5 (4)
C4—N3—C10	116.8 (3)	C15—C11—C10	120.9 (4)
C3—N3—C10	116.0 (3)	C12—C11—C10	122.5 (4)
C2—N2—C3	112.3 (3)	N6—C15—C11	124.9 (4)
C2—N2—Cu1	106.7 (2)	N6—C15—H15	116 (3)
C3—N2—Cu1	114.5 (2)	C11—C15—H15	119 (3)
C2—N2—H2	107 (2)	N6—C14—C13	124.0 (4)
C3—N2—H2	108 (2)	N6—C14—H14	120 (3)
Cu1—N2—H2	108 (2)	C13—C14—H14	116 (3)
C9—N1—C1	112.9 (3)	C14—C13—C12	118.5 (5)
C9—N1—Cu1	115.8 (3)	C14—C13—H13	120 (2)
C1—N1—Cu1	106.8 (2)	C12—C13—H13	122 (2)
C9—N1—H1	109 (3)	C13—C12—C11	120.3 (4)
C1—N1—H1	108 (3)	C13—C12—H12	124 (2)
Cu1—N1—H1	104 (3)	C11—C12—H12	116 (2)
C14—N6—C15	115.9 (4)	C21—C16—C17	118.0 (3)
N5—C6—C5	109.0 (3)	C21—C16—C22	119.9 (3)
N5—C6—H6A	111 (2)	C17—C16—C22	122.1 (3)
C5—C6—H6A	112 (2)	C18—C17—C16	120.7 (3)
N5—C6—H6B	106 (2)	C18—C17—H17	120 (2)
C5—C6—H6B	114 (2)	C16—C17—H17	120 (2)
H6A—C6—H6B	104 (3)	C19—C18—C17	120.6 (4)
N4—C5—C6	109.3 (3)	C19—C18—H18	121 (2)
N4—C5—H5A	110 (2)	C17—C18—H18	119 (2)
C6—C5—H5A	111 (2)	C18—C19—C20	120.0 (3)
N4—C5—H5B	106 (2)	C18—C19—H19	120 (2)
C6—C5—H5B	112 (2)	C20—C19—H19	120 (2)
H5A—C5—H5B	108 (3)	C19—C20—C21	118.9 (3)
N3—C4—N4	115.1 (3)	C19—C20—C23	121.3 (3)
N3—C4—H4A	109 (2)	C21—C20—C23	119.7 (3)
N4—C4—H4A	109 (2)	C16—C21—C20	121.8 (3)
N3—C4—H4B	109 (2)	C16—C21—H21	118 (2)
N4—C4—H4B	106 (2)	C20—C21—H21	121 (2)
H4A—C4—H4B	110 (3)	O2—C22—O1	123.8 (3)
N3—C3—N2	114.3 (3)	O2—C22—C16	117.7 (3)
N3—C3—H3A	107 (2)	O1—C22—C16	118.5 (3)
N2—C3—H3A	105 (2)	O4—C23—O3	124.4 (3)
N3—C3—H3B	108 (2)	O4—C23—C20	119.0 (3)
N2—C3—H3B	107 (2)	O3—C23—C20	116.6 (3)
H3A—C3—H3B	115 (3)	H4WA—O4W—H4WB	104.5
N2—C2—C1	109.5 (3)	H5WA—O5W—H5WB	119.6
N2—C2—H2A	109 (2)		
			
C7—N5—C6—C5	−170.6 (3)	N3—C10—C11—C12	114.6 (4)
Cu1—N5—C6—C5	−39.4 (4)	C14—N6—C15—C11	0.7 (7)
C4—N4—C5—C6	−164.5 (3)	C12—C11—C15—N6	−0.5 (6)
Cu1—N4—C5—C6	−37.7 (3)	C10—C11—C15—N6	178.9 (4)
N5—C6—C5—N4	52.4 (4)	C15—N6—C14—C13	−0.7 (7)
C3—N3—C4—N4	−67.4 (4)	N6—C14—C13—C12	0.4 (7)
C10—N3—C4—N4	74.0 (4)	C14—C13—C12—C11	−0.1 (6)
C5—N4—C4—N3	178.4 (3)	C15—C11—C12—C13	0.1 (6)
Cu1—N4—C4—N3	56.3 (4)	C10—C11—C12—C13	−179.2 (4)
C4—N3—C3—N2	68.6 (4)	C21—C16—C17—C18	−0.1 (5)
C10—N3—C3—N2	−73.2 (4)	C22—C16—C17—C18	177.8 (3)
C2—N2—C3—N3	179.5 (3)	C16—C17—C18—C19	1.3 (6)
Cu1—N2—C3—N3	−58.5 (3)	C17—C18—C19—C20	−1.6 (6)
C3—N2—C2—C1	164.3 (3)	C18—C19—C20—C21	0.6 (5)
Cu1—N2—C2—C1	38.0 (4)	C18—C19—C20—C23	−179.6 (3)
C9—N1—C1—C2	168.2 (3)	C17—C16—C21—C20	−0.8 (5)
Cu1—N1—C1—C2	39.9 (4)	C22—C16—C21—C20	−178.8 (3)
N2—C2—C1—N1	−52.9 (4)	C19—C20—C21—C16	0.6 (5)
C1—N1—C9—C8	177.6 (4)	C23—C20—C21—C16	−179.2 (3)
Cu1—N1—C9—C8	−59.0 (4)	C21—C16—C22—O2	−2.2 (4)
N1—C9—C8—C7	71.4 (5)	C17—C16—C22—O2	179.9 (3)
C6—N5—C7—C8	179.4 (3)	C21—C16—C22—O1	176.0 (3)
Cu1—N5—C7—C8	54.0 (4)	C17—C16—C22—O1	−1.9 (5)
C9—C8—C7—N5	−68.1 (5)	C19—C20—C23—O4	−20.1 (5)
C4—N3—C10—C11	156.2 (3)	C21—C20—C23—O4	159.6 (3)
C3—N3—C10—C11	−62.6 (4)	C19—C20—C23—O3	158.9 (3)
N3—C10—C11—C15	−64.7 (5)	C21—C20—C23—O3	−21.4 (4)

### trans-Diaqua[3-(pyridin-3-ylmethyl)-1,3,5,8,12-pentaazacyclotetradecane-κ4N1,N5,N8,N12]copper(II) isophthalate 0.9-hydrate (II) Hydrogen-bond geometry (Å, º)

**Table:**

*D*—H···*A*	*D*—H	H···*A*	*D*···*A*	*D*—H···*A*
N1—H1···O4	0.79 (4)	2.37 (4)	3.115 (4)	157 (4)
N2—H2···O3^i^	0.81 (3)	2.25 (4)	3.044 (4)	167 (3)
N4—H4···O1^ii^	0.78 (3)	2.31 (3)	3.037 (4)	157 (3)
N5—H5···O2^iii^	0.83 (4)	2.10 (4)	2.910 (4)	163 (3)
O1*W*—H1*WA*···O4^i^	0.85	2.00	2.849 (3)	174
O1*W*—H1*WB*···O2^ii^	0.76	2.07	2.831 (3)	176
O2*W*—H2*WA*···O1^iii^	0.71	2.15	2.859 (3)	178
O2*W*—H2*WB*···O3	0.82	1.90	2.722 (3)	180
O3*W*—H3*WA*···O1^ii^	0.85	1.88	2.731 (6)	179
O3*W*—H3*WB*···O1^iv^	0.85	2.18	2.760 (6)	126
C1—H1*A*···O4^i^	1.05 (4)	2.64 (4)	3.662 (5)	164 (3)
C4—H4*B*···O3*W*^v^	0.98 (4)	2.60 (4)	3.274 (7)	125 (3)
C5—H5*B*···O3*W*^v^	0.94 (4)	2.62 (4)	3.415 (7)	142 (3)
C10—H10*A*···O3*W*	0.92 (4)	2.49 (4)	3.367 (8)	161 (3)
C13—H13···O2^i^	1.03 (4)	2.53 (4)	3.446 (6)	147 (3)
C1—H1*B*···N6^vi^	0.84 (4)	2.66 (4)	3.474 (5)	165 (4)

Symmetry codes: (i) *x*+1, *y*, *z*; (ii) *x*+1, −*y*+1/2, *z*+1/2; (iii) *x*, −*y*+1/2, *z*+1/2; (iv) −*x*+1, *y*−1/2, −*z*+1/2; (v) −*x*+1, −*y*, −*z*+1; (vi) −*x*+1, *y*+1/2, −*z*+1/2.
